# Supplementary material for: CALCOCO1 acts with VAMP‐associated proteins to mediate ER‐phagy
Source: EMBO J. 2020 Jun 11;39(15):e103649. doi: 10.15252/embj.2019103649 (PMC7396842; doi:10.15252/embj.2019103649)

Appendix: Table of Contents

Appendix Table S1…………………………………page 2-7

Appendix Figure legends………………. …………page 8

Appendix Figure S1…………………….………..page 9

Appendix Figure S2……………………………….. page 10

Appendix Table S1. **Plasmids used in this study**

pDONOR221-CALCOCO1	This study
pDONOR221-CALCOCO1 141-LVV/141-AAA	This study
pDONOR221-CALCOCO1 (1-150)	This study
pDONOR221-CALCOCO1(145-513)	This study
pDONOR221-CALCOCO1(514-691)	This study
pDONOR221-CALCOCO1ΔSKICH	This study
pDONOR221-CALCOCO1ΔCC	This study
pDONOR221-CALCOCO1Δ514-691	This study
pDONOR221-CALCOCO1ΔLIRΔ514-691	This study
pDONOR221-CALCOCO1ΔLIRΔ623-691	This study
pDONOR221-CALCOCO1Δ145-205	This study
pDONOR221-CALCOCO1Δ231-339	This study
pDONOR221-CALCOCO1Δ413-513	This study
pDONOR221-CALCOCO1(145-670)	This study
pDONOR221-CALCOCO1(145-680)	This study
pDONOR221-CALCOCO1(680-FFF/AAA)	This study
pDONOR221-CALCOCO1Δ671-691	
pDest-MYC-CALCOCO1	This study
pDest-MYC-CALCOCO1ΔLIR	This study
pDest-MYC-CALCOCO1 (1-150)	This study
pDest-MYC-CALCOCO1-(145-513)	This study

pDest-MYC-CALCOCO1(514-691)	This study
pDest-MYC-CALCOCO1ΔSKICHΔLIR	This study
pDest-MYC-CALCOCO1ΔCC	This study
pDest-MYC-CALCOCO1Δ514-691	This study
pDest-MYC-CALCOCO1ΔLIRΔ623-691	This study
pDest-MYC-CALCOCO1Δ145-205	This study
pDest-MYC-CALCOCO1Δ231-339	This study
pDest-MYC-CALCOCO1Δ413-513	This study
pDest-MYC-CALCOCO1(145-670)	This study
pDest-MYC-CALCOCO1(145-680)	This study
pDest-MYC-CALCOCO1Δ(680-FFF/AAA)	This study
PDest53-CALCOCO1	This study
pcDNA-Dest53 (GFP fusion)	Invitrogen
pDest-EGFP-C1	Lamark et al., 2003
pDest-EGFP-CALCOCO1	This study
pDest-EGFP-CALCOCO1Δ671-691	This study
pDest-FlpIn-EGFP-CALCOCO1	This study
pDestmCherry-CALCOCO1	This study
pDONOR221-TAX1BP1	This study
pDONOR221-TAX1BP1ΔLIR	This study
pDONOR221-TAX1BP1mLIRΔ701-789	This study
pDest-MYC-TAX1BP1	This study

pDest-MYC-TAX1BP1ΔLIR	This study
pDest-MYC-TAX1BP1mLIRΔ701-789	This study
pDONOR221-NDP52	This study
pDONOR221-NDP52ΔLIR	This study
pDest-MYC-NDP52	This study
pDest-MYC-NDP52ΔLIR	This study
pDONOR221-VAPA	This study
pDONOR221-VAPA(K94D/M96D)	This study
pDest-MYC-VAPA	This study
pDest-FlpIn-EGFP-VAPA	This study
PDest15-VAPA	This study
PDest15-VAPA(K94D/M96D)	This study
pDest-mCherry-EYFP-VAPA	This study
pDONOR221-VAPB	This study
pDONOR221-VAPB(K87D/M89D)	This study
PDest15-VAPB	This study
pDest-MYC-VAPB	This study
pDest15-VAPB(K87D/M89D)	This study
pENTR-GABARAP	Pankiv et al., 2007
pENTR-GABARAPL1	Pankiv et al., 2007
pENTR-GABARAPL2	Pankiv et al., 2007
pENTR-LC3A	Pankiv et al., 2007

pENTR-LC3B	Pankiv et al., 2007
pENTR-LC3C	Kirkin et al., 2009
pENTR-GABARAP Y49A (mLDS)	Alemu et al., 2012
pENTR-GABARAPL1 Y49A (mLDS)	This study
pENTR-GABARAPL2 Y49A (mLDS)	This study
pENTR-LC3B F52A (mLDS)	Kirkin et al., 2009
pENTR-LC3C F58A (mLDS)	This study
pENTR-GABARAP ΔUDS	This study
pENTR-GABARAPL1 ΔUDS	This study
pENTR-GABARAPL2 ΔUDS	This study
pENTR-GABARAP Y49A/ΔUDS	This study
pENTR-GABARAPL1 Y49A/ΔUDS	This study
pENTR-GABARAPL2 Y49A/ΔUDS	This study
pENTR-LC3B F52A/ΔUDS	This study
pENTR-LC3C F58A/ΔUDS	This study
pDest15-GABARAP	Pankiv et al., 2007
pDest15-GABARAPL1	Pankiv et al., 2007
pDest15-GABARAPL2	Pankiv et al., 2007
pDest15-LC3A	Pankiv et al., 2007
pDest15-LC3B	Pankiv et al., 2007
pDest-mCherry-EYFP-LC3B	Pankiv et al., 2007
pDest15-LC3C	Kirkin et al., 2009

pDest15-GABARAP Y49A	Alemu et al., 2012
pDest15-GABARAPL1 Y49A	This study
pDest15-GABARAPL2 Y49A	This study
pDest15-LC3B F52A	Kirkin et al., 2009
pDest15-LC3C F58A	This study
pDest15-GABARAP ΔUDS	This study
pDest15-GABARAPL1 ΔUDS	This study
pDest15-GABARAPL2 ΔUDS	This study
pDest15-GABARAP Y49A/ΔUDS	This study
pDest15-GABARAPL1 Y49A/ΔUDS	This study
pDest15-GABARAPL2 Y49A/ΔUDS	This study
pDest15-LC3B F52A/ΔUDS	This study
pDest15-LC3C F58A/ΔUDS	This study
pCDH EF1alfa IRES Puro mCherry EGFP FAM13 4b 82-238	This study

References:

Alemu, E.A.,, Lamark, T., Torgersen, K.M., Birgisdottir, A.B., Bowitz Larsen, K., Jain, A., Olsvik, H., Øvervatn, A., Kirkin, V. and Johansen, T. 2012. ATG8 Family Proteins Act as Scaffolds for Assembly of the ULK Complex: Sequence Requirements for LC3-Interacting Region (LIR) Motifs. ***J. Biol. Chem.*** **287,** 39275-39290

Kirkin, V., Lamark, T., Sou, Y.S., Bjørkøy, G., Nunn, J.L., Bruun J-A., Shvets, E., McEwan, D.G., Clausen, T.H., Wild, P., Bilusic, I., Theurillat, J.P., Øvervatn, A., Ishii, T., Elazar, Z., Komatsu, M., Dikic, I. and Johansen, T. 2009. A role for NBR1 in autophagosomal degradation of ubiquitinated substrates. ***Mol. Cell*** **33,** 505-516.

Lamark, T., Perander, M., Outzen, H., Kristiansen, K., Øvervatn, A., Michaelsen, E., Bjørkøy, G. and Johansen, T. 2003. Interaction codes within the family of mammalian PB1 domain-containing

proteins. ***Journal of Biological Chemistry,*** **278,** 34568-34581.

Pankiv, S., Høyvarde Clausen, T., Lamark, T., Brech, A., Bruun, J-A., Outzen, H., Øvervatn, A., Bjørkøy, G. and Johansen, T. 2007. p62/SQSTM1 binds directly to Atg8/LC3 to facilitate degradation of ubiquitinated protein aggregates by autophagy. ***Journal of Biological Chemistry,*** **282,** 24131-24145.

Appendix Figure S1. Generation of CRISPR/CAS9-mediated HeLa and HEK293 FlpIn CALCOCO1 KO cells,

A Hela FlpIn CALCOCO1 KO cells generated with the indicated gRNA for genomic editing of targeted exons 2. Shown are the genomic sequences of four mutated alleles in CALCOCO1 KO clone 38.

B HEK293 FlpIn CALCOCO1 KO cells generated targeting exon 2 and exon 4 with two different gRNAs. CALCOCO1 KO cells were verified with both western blot and genomic sequencing of regions encompassing exon 2 and exon 4.

Appendix Figure S2. Co-localization of CALCOCO1 with p62 and LC3B depends on the LIR and UIR motifs.

A, B HeLa CALCOCO1 KO cells reconstituted with EGFP-CALCOCO1 (A) or EGFP-CALCOCO1 mLIR+ΔUIR (B) were starved with or without Baf A1 treatment as indicated, and then immunostained for endogenous p62 and LC3B. Scale bars, 5 μm.

C HeLa CALCOCO1 KO cells stably expressing EGFP-CALCOCO1 were starved or not for 2 hours and then immunostained with anti-ATG13 and anti-WIPI2 antibodies. Scale bars, 10 μm.

Appendix Fig S1

A

CRISPR/CAS9 CALCOCO1 KO in HeLa FlpIn cells

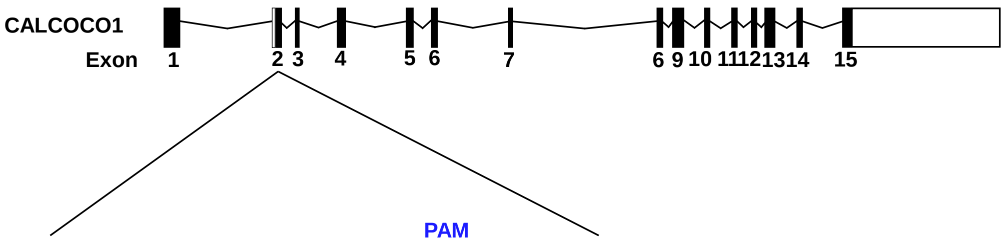


```
                                 PAM
AGGCCAGGATGGAAGAATCACCACTAAGCCGGGCACCATCCCG
TCCGGTCCTACCTTCTTAGTGGTGATTCGGCCCGTGGTAGGGC
```

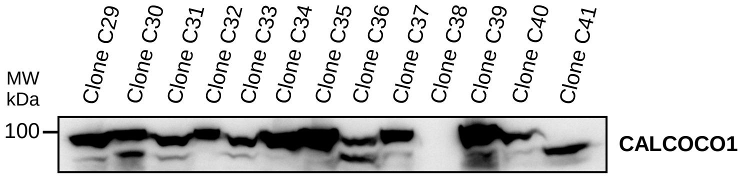


```
CALCOCO1  WT  AGGCCAGGATGGAAGAATCACCACTAA-GCCGGGCACCATCCCG  43
CALCOCO1 C38  AGGCCAGGATGGAAGAATCACCACTAAAGCCGGGCACCATCCCG  44
CALCOCO1 C38  AGGCCAGGATGGAAGAATCACCACTCAAGTCAGAGGTGGCGAAA
              CCCGACAGGACTATAAAGATACCAGGCGTTTCCCCCTGGAAGCT
              CCCTCGTGCGCTCTCCTGTTCCGACCCTGCCGCTTACCGGATAC
              CTGTCCGCCTTTCTCCCTTCGGGAAGCGTGGCGCTTTCTCATAG
              CTCACGCTGTAGGTATCTCAGTTCGGTGTAGGTCGTTCGCTCCA
              AGCCGGGCACCATCCCG                            237
CALCOCO1 C38  AGGCCAGGATGGAAGAATCACCACTGCGCCCAATACGCAAACCG
              CCTCTCCCCGCGCGTTGGCCGATTCATTAATGCAGCTGGCACGA
              CAGGTTTCCCGACTGGAAAGCGGGCAGTGAGCGCAACGCAATTA
              ATGTGAGTTAGCTCACTCATTAGGCACCCCAGGCTTTACACTTT
              ATGCTTCCGGCTCGTATGTTGTGTGGAATTGTGAGCGGATAACA
              ATTTCACACAGGAAACAGCTATGACCATGATTACGCCGGGCACC
              ATCCCG                                       270
CALCOCO1 C38  AGGCCAGGATGGAAGAATCACCACTATACGCAAACCGCCTCTCC
              CCGCGCGTTGGCCGATTCATTAATGCAGCTGGCACGACAGGTTT
              CCCGACTGGAAAGCGGGCAGTGAGCGCAACGCAATTAATGTGAG
              TTAGCTCACTCATTAGGCACCCCAGGCTTTACACTTTATGCTTC
              CGGCTCGTATGTTGTGTGGAATTGTGAGCGGATAACAATTTCAC
              ACAGGAAACAGCTATGACCATGATTACGCCAAGCTATTTAGGTG
              ACACTATAGAATACTCAAGCTATGCATCCAACGCGTTGGGAGCT
              CTCCCATATGGTCGACCTGCAGGCGGCCGCGAATTCACTAGTGA
              TTCTCACAGCCCATAAACCTAAGCCGGGCACCATCCCG       390
```

B

CRISPR/CAS9 CALCOCO1 KO in HEK293 FlpIn cells

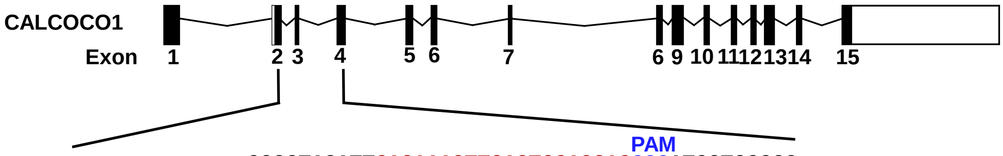


```
                                                     PAM
Exon 2 Target 1  GGGCTACATTGAGAAAGTTGACTCCACCACGGGATGGTGCCCG
                 CCCGATGTAACTCTTTCAACTGAGGTGGTGCCCTACCACGGGC
                                                     PAM
Exon 4 Target 2  AGCTCTACCAGTTCCGATATGTGAACCGCCAGGGCCAGGTGTG
                 TCGAGATGGTCAAGGCTATACACTTGGCGGTCCCGGTCCACAC
```

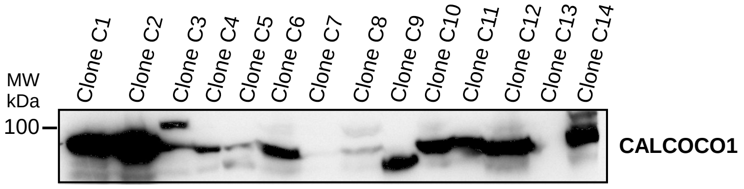

Appendix Fig S2

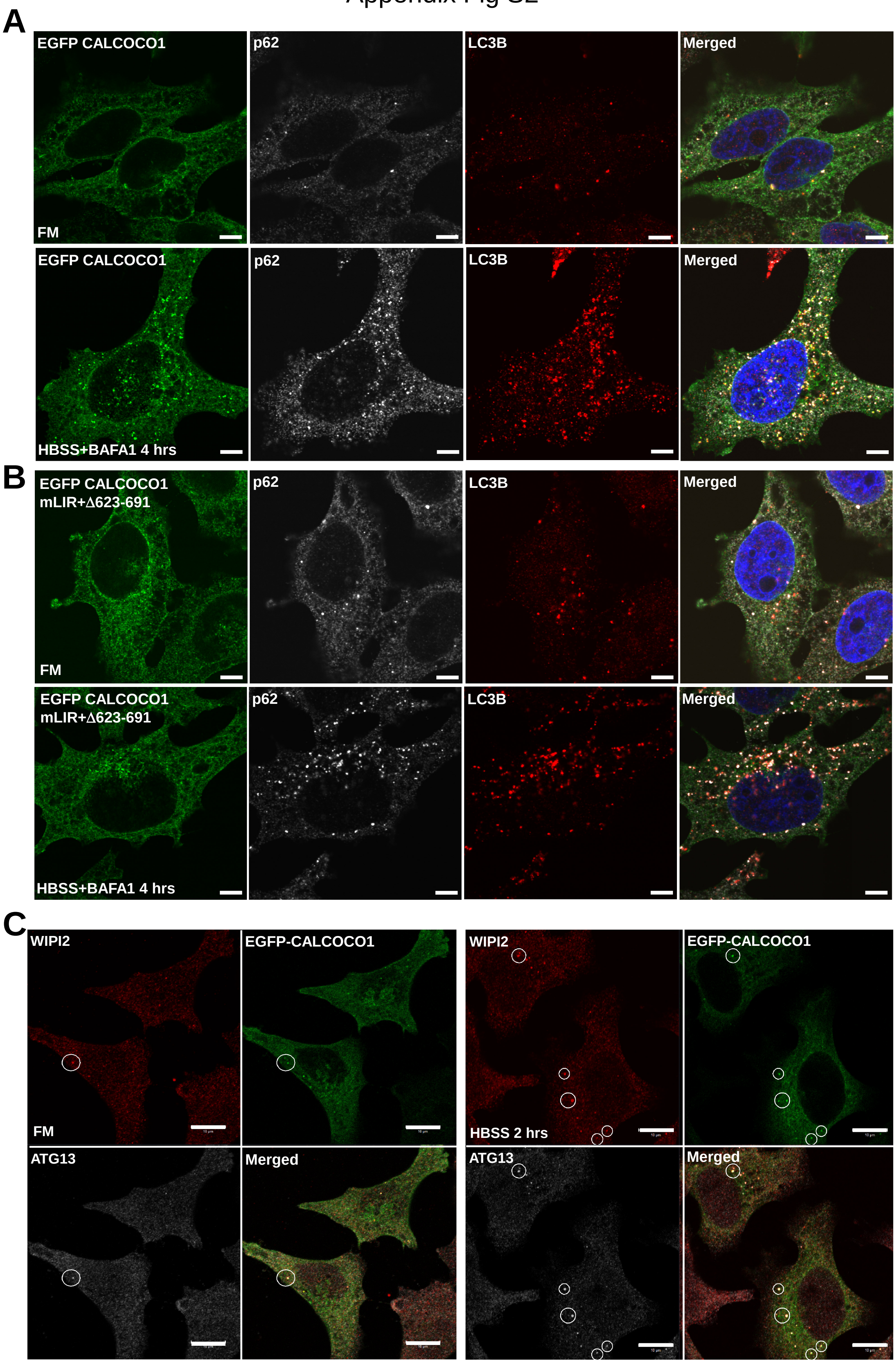

Supplement: Supplementary file 1 — Appendix [file EMBJ-39-e103649-s001.pdf]
