# Supplementary material for: CALCOCO1 acts with VAMP‐associated proteins to mediate ER‐phagy
Source: EMBO J. 2020 Jun 11;39(15):e103649. doi: 10.15252/embj.2019103649 (PMC7396842; doi:10.15252/embj.2019103649)
Supplement: Supplementary file 2 — Expanded View Figures PDF [file EMBJ-39-e103649-s002.pdf]

Expanded View Figures

Figure EV1. CALCOCO1 homomerizes via coiled-coil domains, but does not heterodimerize with TAX1BP1 or NDP52.

A Co-immunoprecipitation (co-IP) of Myc-CALCOCO1 with EGFP-CALCOCO1, following transient co-transfection of EGFP-CALCOCO1 and indicated Myc-CALCOCO1 constructs in HEK293 cells.

B Co-IP of Myc-CALCOCO1 with EGFP-CALCOCO1. ^{35}S-GFP-CALCOCO1 (upper panels) or ^{35}S-GFP (lower panels) were *in vitro* co-transcribed/translated with indicated ^{35}S-Myc-CALCOCO1 constructs. GFP-CALCOCO1 or GFP, respectively, were immunoprecipitated with GFP-TRAP and the immunoprecipitates then resolved by SDS–PAGE. The resolved immunoprecipitates were detected by autoradiography.

C GFP-CALCOCO1 or GFP were *in vitro* co-transcribed/translated with Myc-CALCOCO1, Myc-NDP52, or Myc-TAX1BP1 and then immunoprecipitated with GFP-TRAP. The analysis of the immunoprecipitates was done as in B. GFP construct is indicated with a circle and Myc-constructs with a star.

D GST pull-down analyses of binding of *in vitro* transcribed/translated ^{35}S-Myc-CALCOCO1 with recombinant GST-tagged Galectin-3 and-8. *In vitro* transcribed/translated Myc-NDP52 and Myc-TAX1BP1 were included as positive controls.

© 2020 The Authors

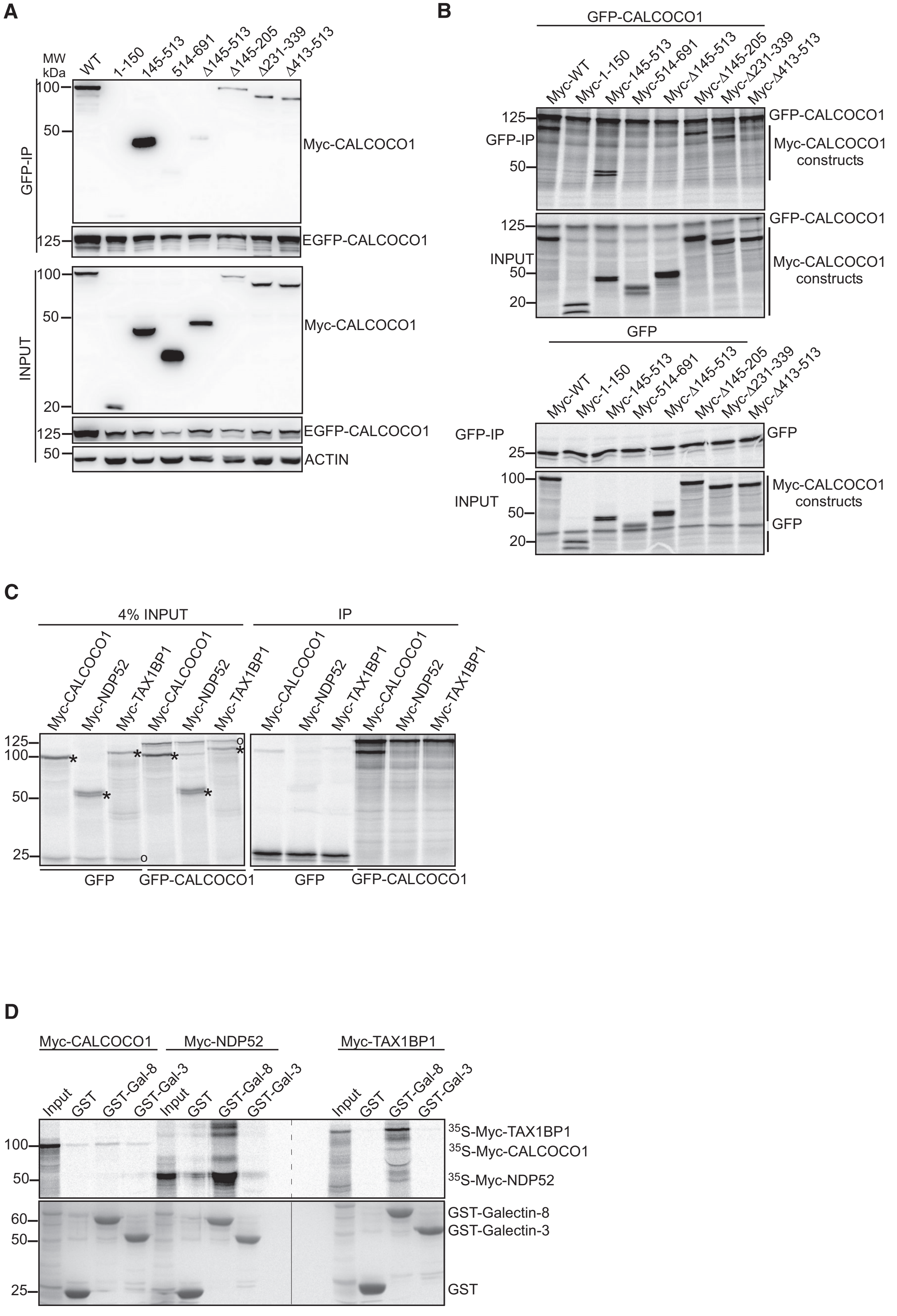

Figure EV1.

 © 2020 The Authors

Figure EV2. CALCOCO1 is degraded by autophagy and co-localizes in HBSS-treated cells with p62, LC3B, and GABARAP.

A, B Immunoblot analysis of indicated cell lines, starved for 6 h (HBSS) as indicated, and treated with MG132 or Baf A1 as indicated.

C Extension of Fig 1G. HeLa CALCOCO1 KO cells stably transfected with EGFP-CALCOCO1 were induced with tetracycline for 24 h and then starved (HBSS) with or without Baf A1 treatment as indicated, before immunostaining for endogenous p62 and LC3B. Scale bars, 5 μm for confocal microscopy images, 2 μm for airyscans.

D Extension of Fig 1J. Percentage of LAMP1 rings associated with a CALCOCO1 structure. The error bars represent mean ± SEM of three independent experiments per condition and 200 cells per experiment.

E HeLa cells stably transfected with EGFP-CALCOCO1 were treated with tetracycline for 24 h to induce expression of EGFP-CALCOCO1. Cells were then starved or not and immunostained with anti-p62, anti-LC3, and anti-GABARAP antibodies as indicated. Co-localization in dots is indicated by circles and supported using line plots shown below the micrographs. Scale bars, 10 μm.

© 2020 The Authors

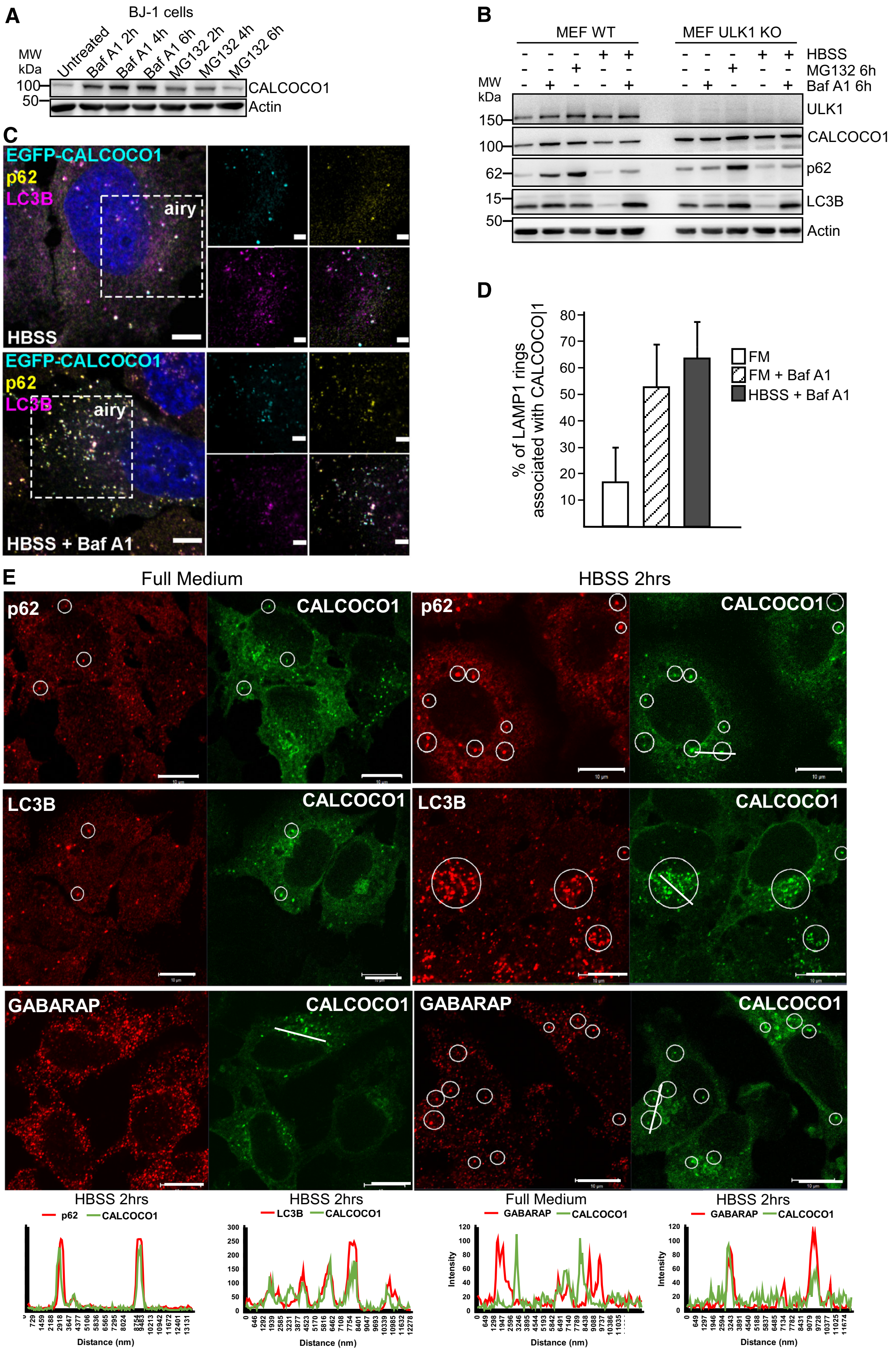

Figure EV2.

 © 2020 The Authors

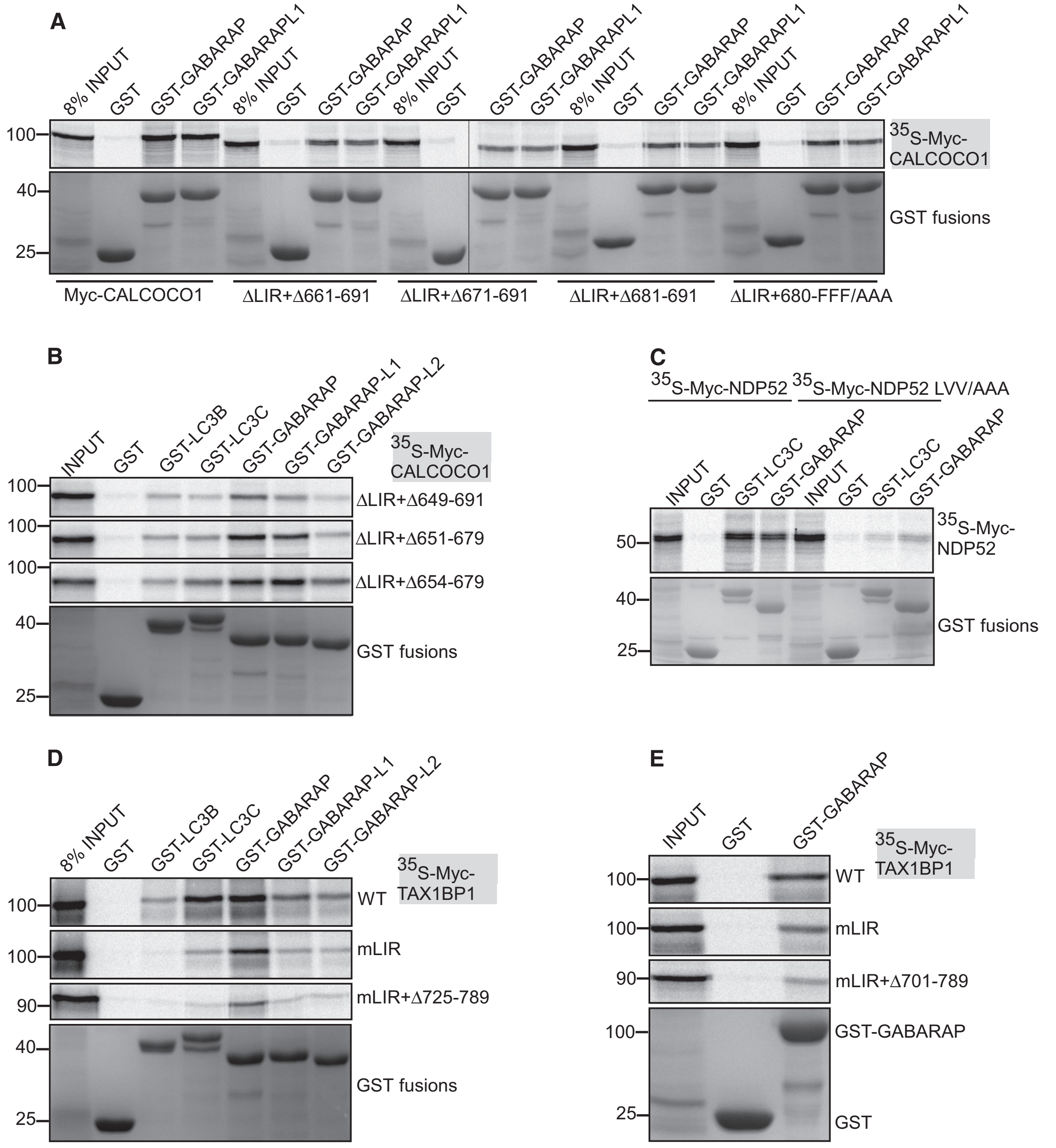

Figure EV3. The C-terminal parts of CALCOCO1 and TAX1BP1 contribute to their interaction with ATG8 family proteins.

A, B GST pull-down binding of the indicated *in vitro* transcribed/translated ^{35}S-Myc-CALCOCO1 constructs with recombinant GST-tagged ATG8 family proteins.

C GST pull-down analyses of binding of *in vitro* transcribed/translated WT or LIR-mutated (LVV/AAA) ^{35}S-Myc-NDP52 with recombinant GST-tagged ATG8 family proteins.

D, E GST pull-down analyses of binding of indicated *in vitro* transcribed/translated ^{35}S-Myc-TAX1BP1 constructs with indicated recombinant GST-tagged ATG8 family proteins.

© 2020 The Authors

Figure EV4. CALCOCO1 KO inhibits basal autophagy.

A, B WT or CALCOCO1 KO HeLa cells were transiently transfected with mCherry-EGFP-LC3B and 24 h after transfection, the cells were treated or not with HBSS as indicated. In (A), representative confocal images are shown, and in (B), the fraction of red-only puncta is counted and shown as a percentage. The error bars represent mean ± SD of red-only puncta percentages of three independent experiments. Statistical comparison was analyzed by one-way ANOVA followed by Tukey multiple comparison test and significance displayed as ***$P < 0.001$; ns is not significant. The arrows in FM show either red-only or red + green puncta while in the
HBSS-treated cells, the arrows show red-only puncta.

C GST pull-down analyses of binding of different *in vitro* transcribed/translated ^{35}S-Myc-tagged ER proteins to recombinant GST-tagged CALCOCO1. GST is included as a negative control.

D CALCOCO1 KO HeLa cells were transiently transfected with the indicated EGFP-CALCOCO1 constructs and immunostained for endogenous VAPA. Scale bars, 10 μm.

Source data are available online for this figure.

 © 2020 The Authors

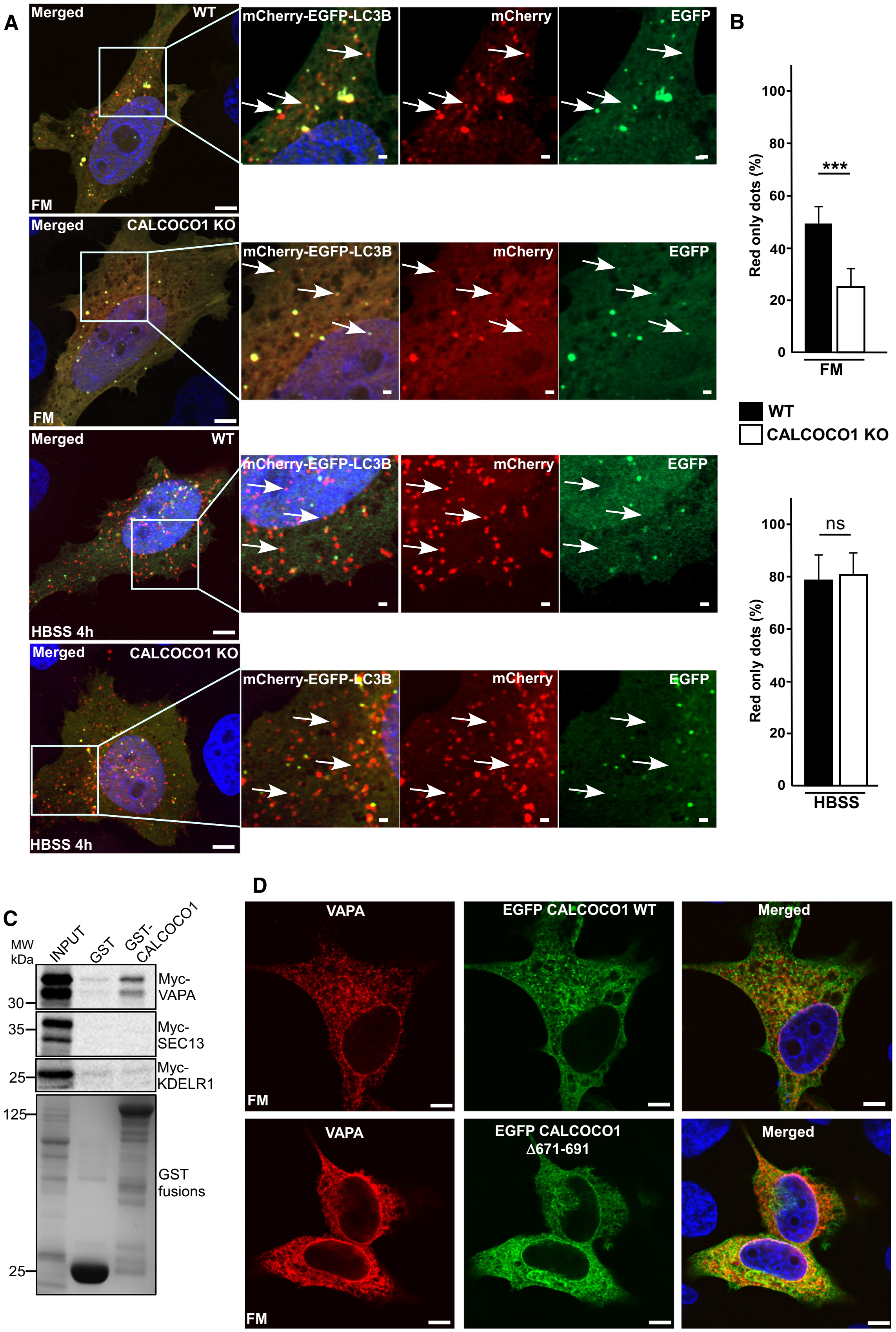
A
Merged
WT
FM
mCherry-EGFP-LC3B
mCherry
EGFP
CALCOCO1 KO
HBSS 4h
B
Red only dots (%)
***
ns
FM
HBSS
WT
CALCOCO1 KO
C
MW kDa
INPUT
GST
GST-CALCOCO1
Myc-VAPA
Myc-SEC13
Myc-KDELR1
GST fusions
D
VAPA
EGFP CALCOCO1 WT
EGFP CALCOCO1 Δ671-691
Merged

Figure EV4.

Figure EV5. CALCOCO1 KO inhibits autophagic degradation of VAPA under starvation.

A, B WT or CALCOCO1 KO HeLa cells were transiently transfected with mCherry-EGFP-VAPA or mCherry-EGFP-FAM134B as indicated and 24 h after transfection, the cells were treated or not with HBSS as indicated. In (A), representative confocal images are shown, and in (B), the fraction of red-only puncta is counted and shown as a percentage. The error bars represent mean ± SD of red-only puncta percentages of three independent experiments. Statistical comparison was analyzed by one-way ANOVA followed by Tukey multiple comparison test and significance displayed as $^{***}P < 0.001$; ns is not significant. The arrows in FM show either red-only (WT cells) or red + green puncta (CALCOCO1 KO cells) while in the HBSS-treated cells, the arrows show red-only puncta.

C Immunoblot analysis of HeLa CALCOCO1 KO cell lines reconstituted with EGFP-CALCOCO1 Δ145–513. Expression of EGFP-CALCOCO1 Δ145–513 was induced or not with tetracycline, and the cells were treated with MG132, HBSS, or Baf A1 as indicated.

Source data are available online for this figure.

© 2020 The Authors

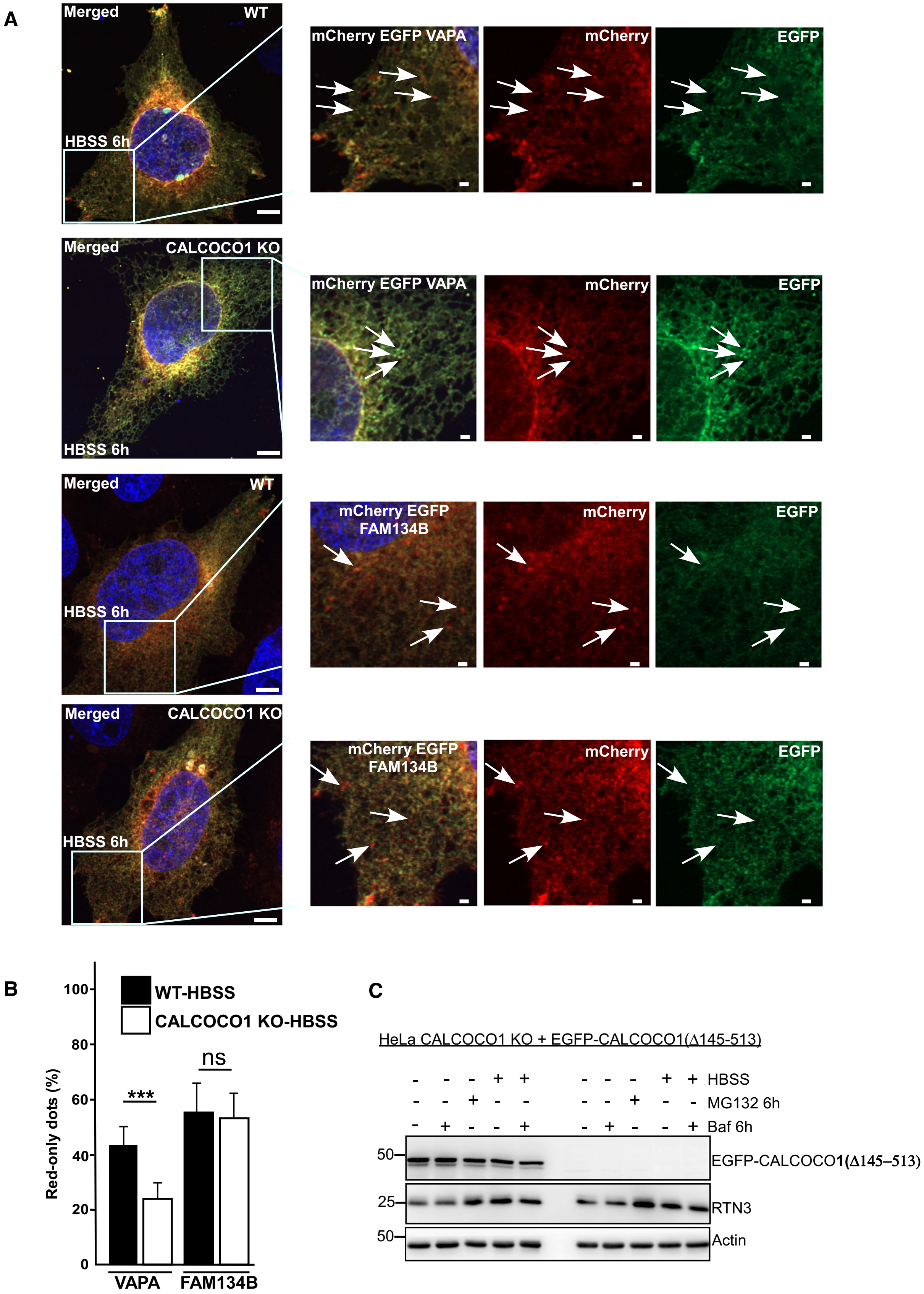
A
Merged
WT
HBSS 6h
mCherry EGFP VAPA
mCherry
EGFP
Merged
CALCOCO1 KO
HBSS 6h
mCherry EGFP VAPA
mCherry
EGFP
Merged
WT
HBSS 6h
mCherry EGFP FAM134B
mCherry
EGFP
Merged
CALCOCO1 KO
HBSS 6h
mCherry EGFP FAM134B
mCherry
EGFP
B
WT-HBSS
CALCOCO1 KO-HBSS
Red-only dots (%)
100
80
60
40
20
0
***
ns
VAPA
FAM134B
C
HeLa CALCOCO1 KO + EGFP-CALCOCO1(Δ145-513)
- - - + + - - - + + HBSS
- - + - - - - + - - MG132 6h
- + - - + - + - - + Baf 6h
50
EGFP-CALCOCO1(Δ145–513)
25
RTN3
50
Actin

Figure EV5.
