# Supplementary figures and images for: CALCOCO1 acts with VAMP‐associated proteins to mediate ER‐phagy
Source: EMBO J. 2020 Jun 11;39(15):e103649. doi: 10.15252/embj.2019103649 (PMC7396842; doi:10.15252/embj.2019103649)

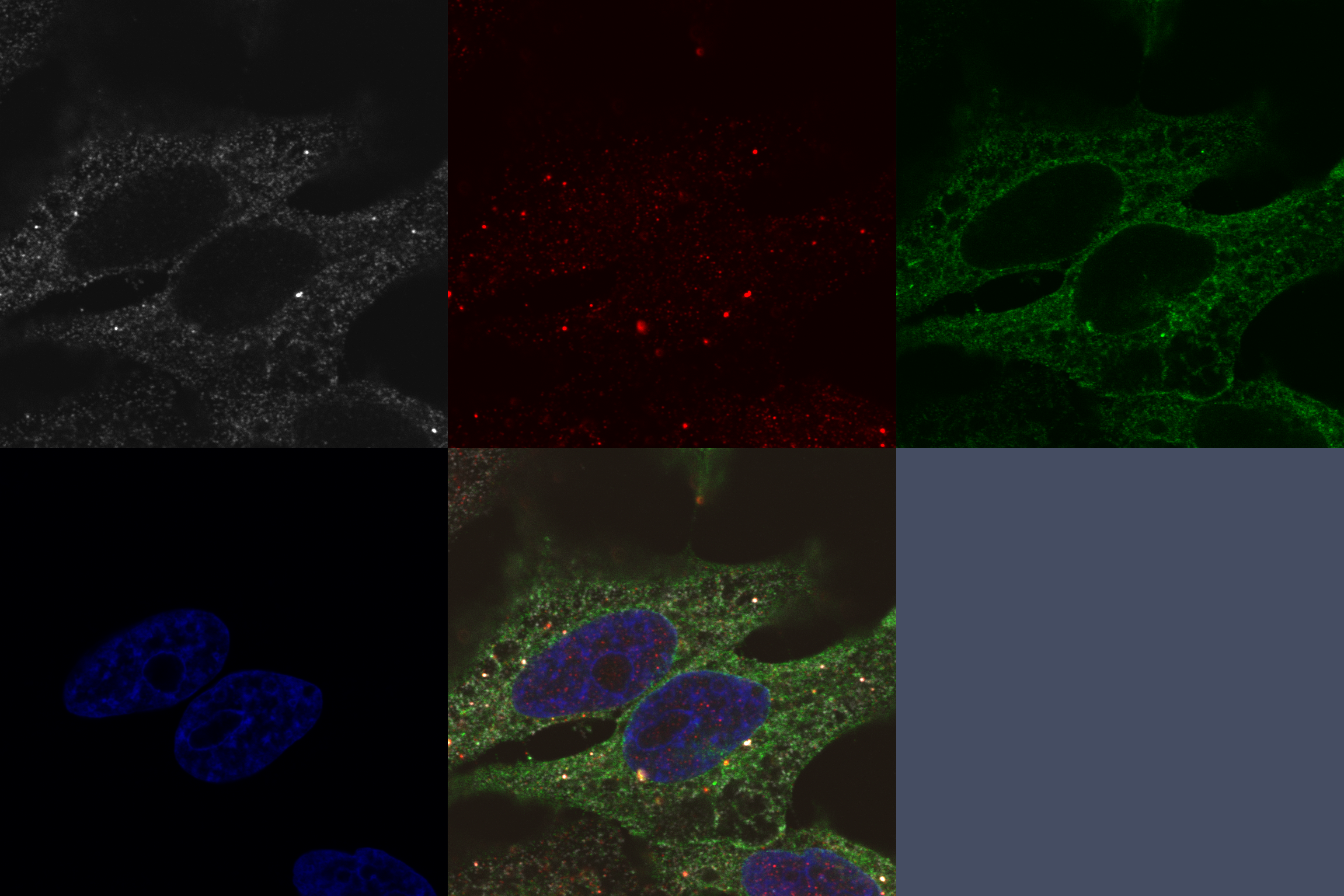

Supplement: Supplementary file 3 — Source Data for Expanded View [file EMBJ-39-e103649-s007.zip › EV_Figure_Source_Data/FigureEV5/Figure_EV5A_upper_panel_Source_Data-sd.tif]

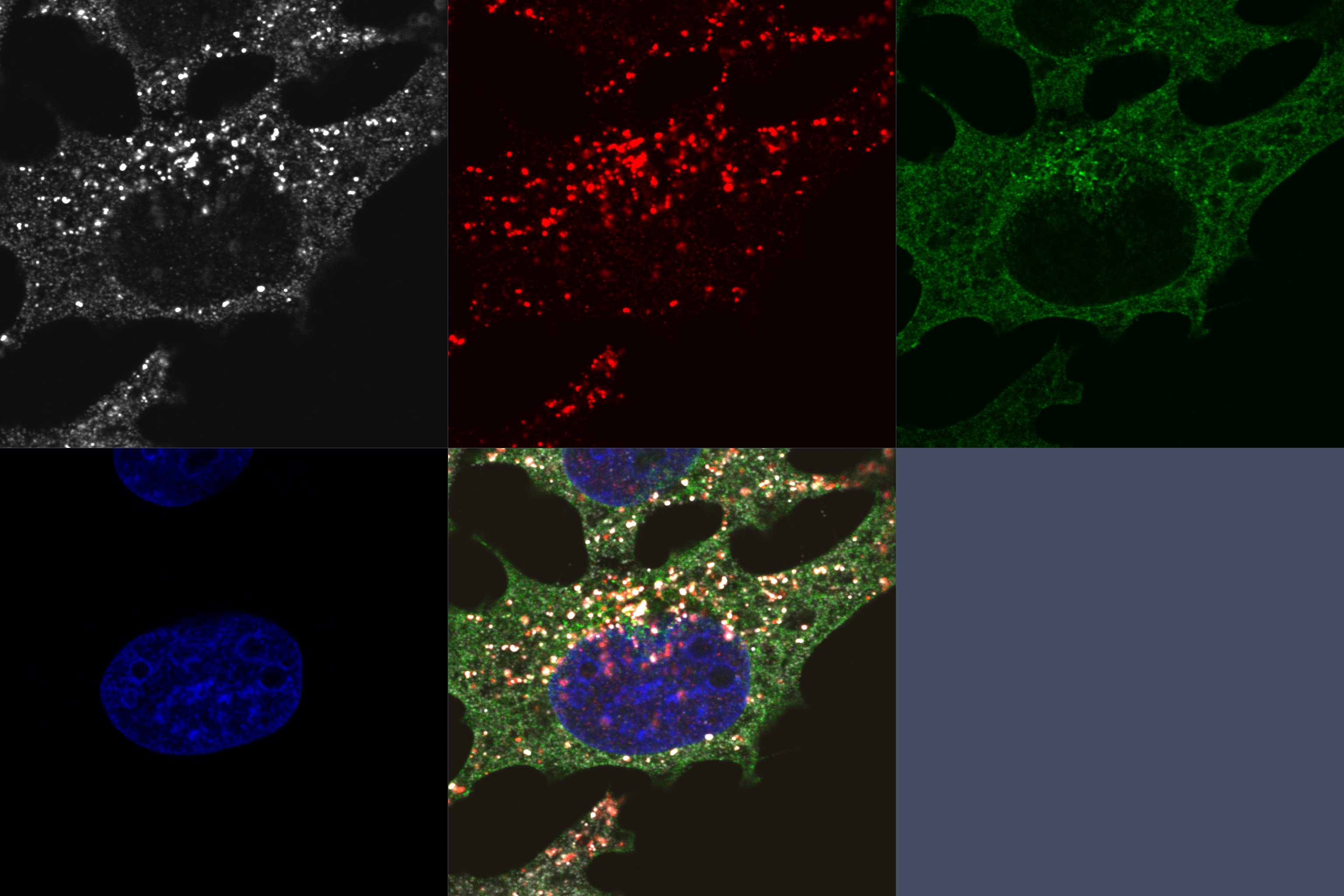

Supplement: Supplementary file 3 — Source Data for Expanded View [file EMBJ-39-e103649-s007.zip › EV_Figure_Source_Data/FigureEV5/Figure_EV5B_lower_panel_Source_Data-sd.tif]

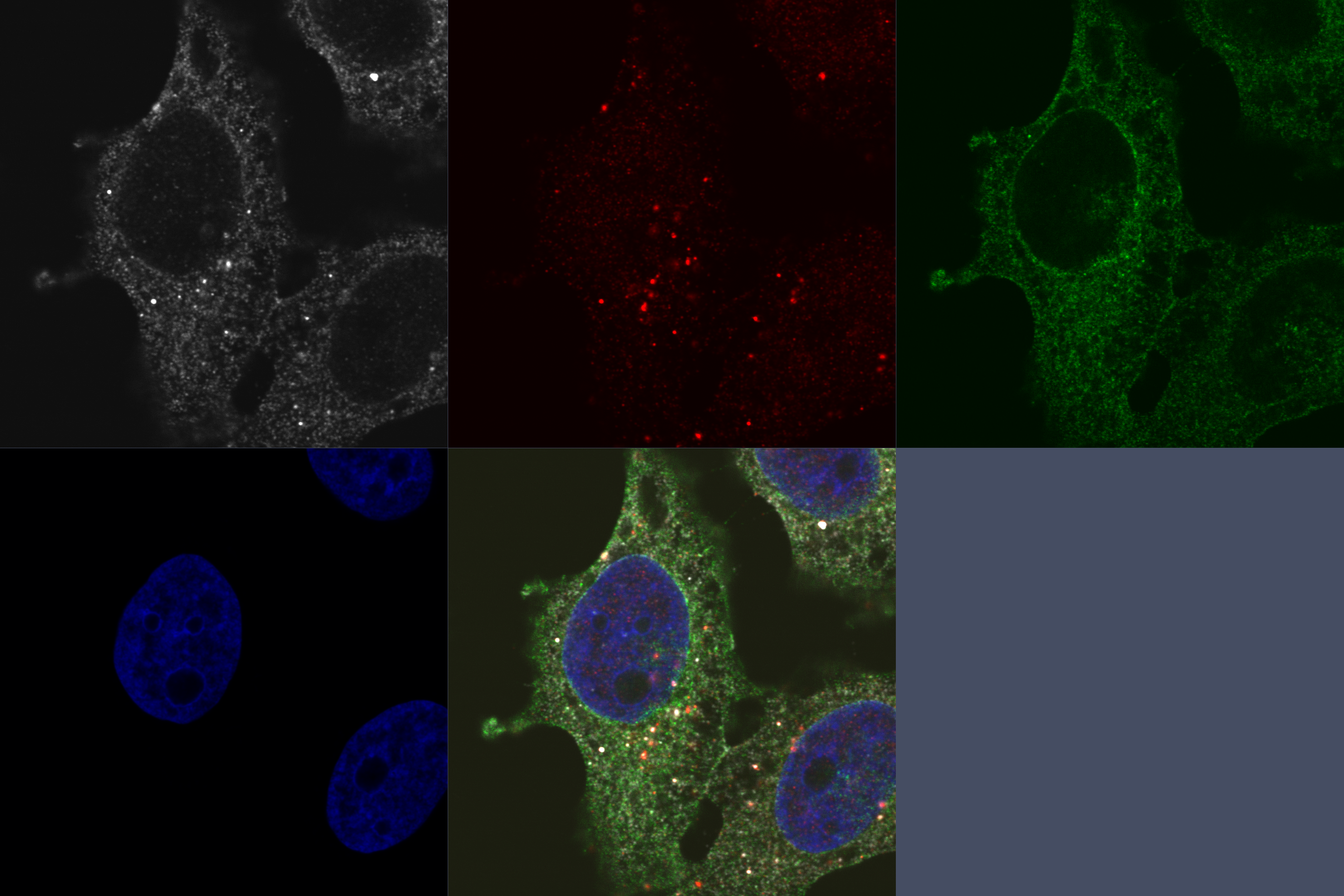

Supplement: Supplementary file 3 — Source Data for Expanded View [file EMBJ-39-e103649-s007.zip › EV_Figure_Source_Data/FigureEV5/Figure_EV5B_upper_panel_Source_Data-sd.tif]

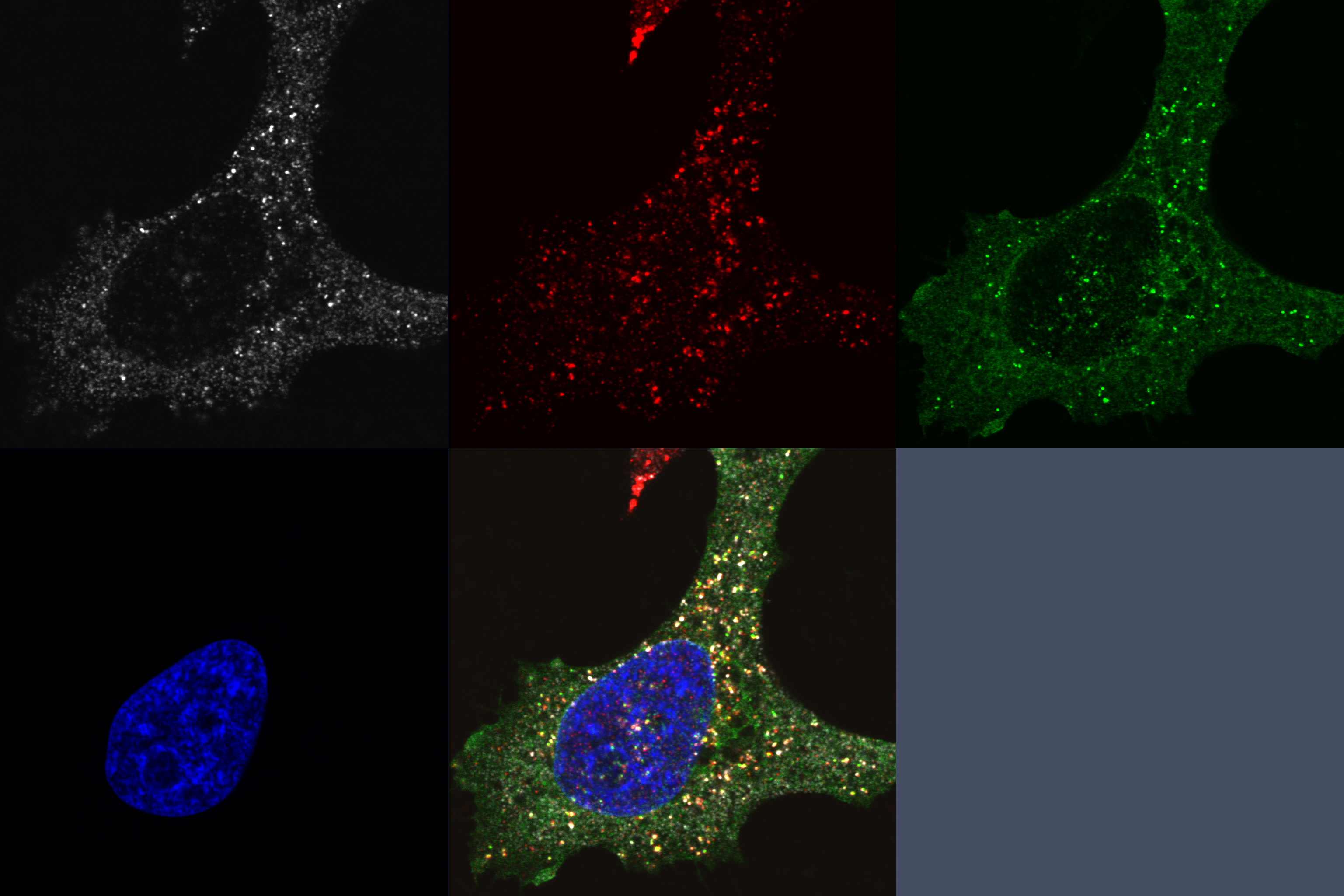

Supplement: Supplementary file 3 — Source Data for Expanded View [file EMBJ-39-e103649-s007.zip › EV_Figure_Source_Data/FigureEV5/Figure_EV5A_lower_panel_Source_Data-sd.tif]

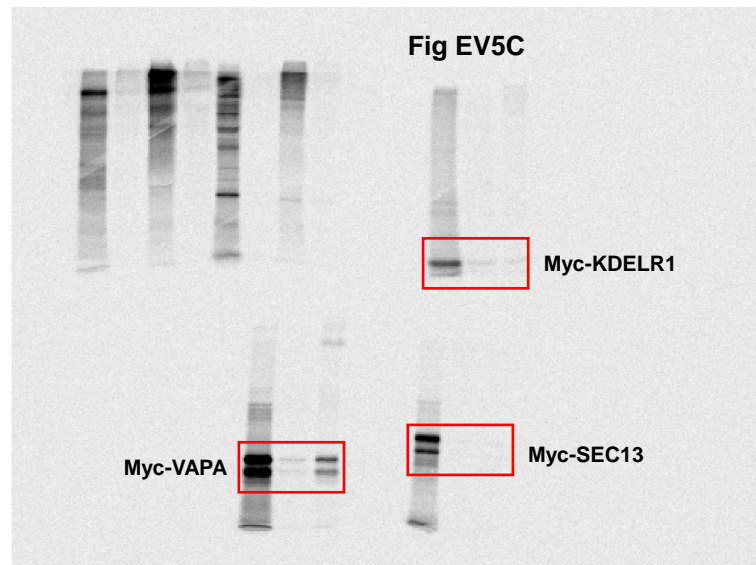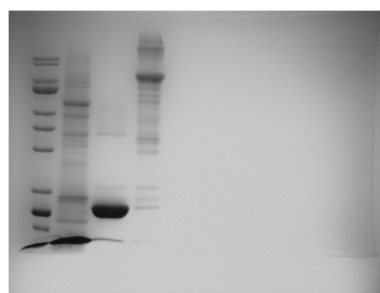

***Coomassie***

Supplement: Supplementary file 3 — Source Data for Expanded View [file EMBJ-39-e103649-s007.zip › EV_Figure_Source_Data/Figure_EV4C_Source_Data-sd.pdf]

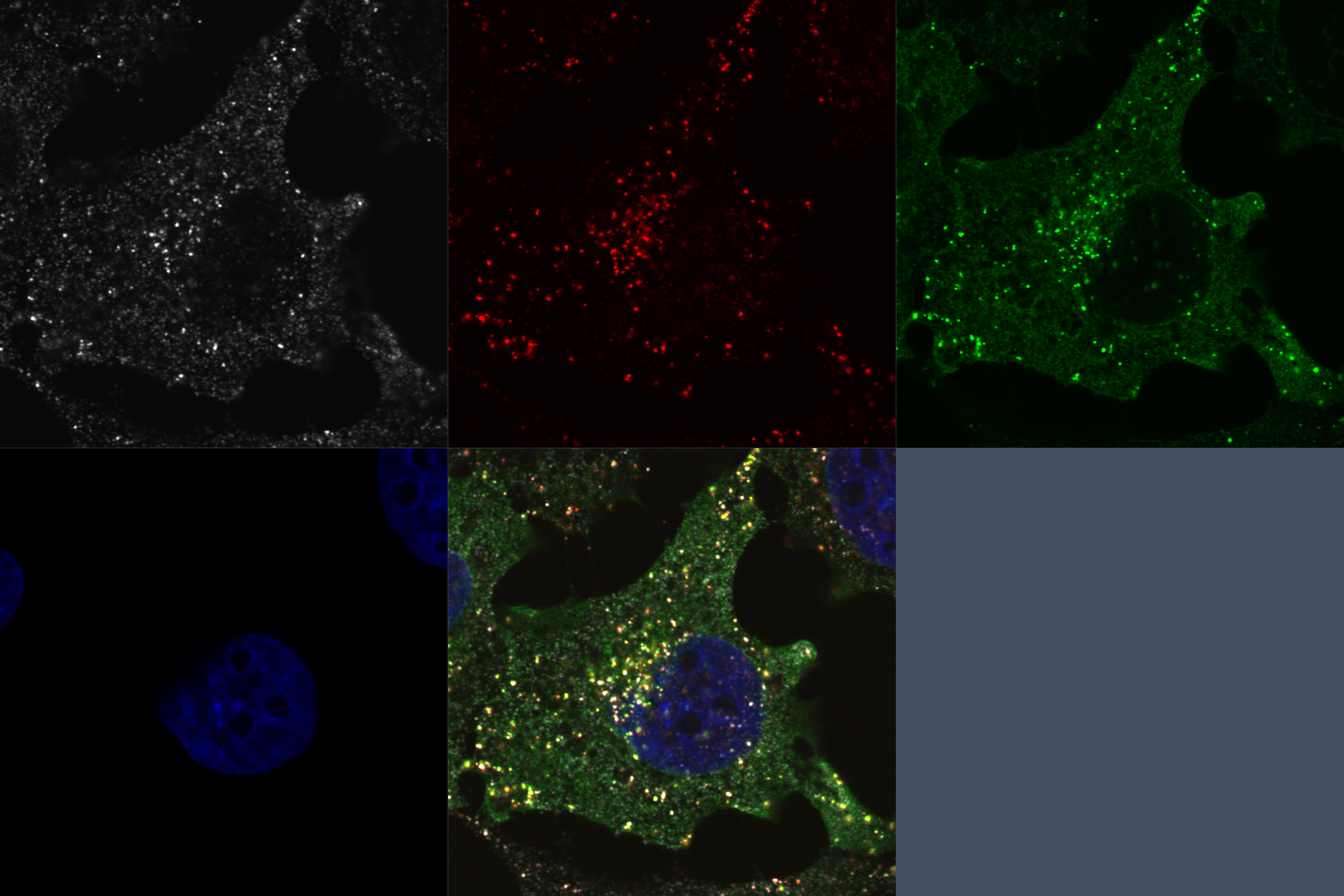

Supplement: Supplementary file 5 — Source Data for Figure 3 [file EMBJ-39-e103649-s003.zip › Figure3_Source_Data/EMBOJ-2019-103649R2-Figure_3G_left_hand_panel_Source_Data-sd.tif]

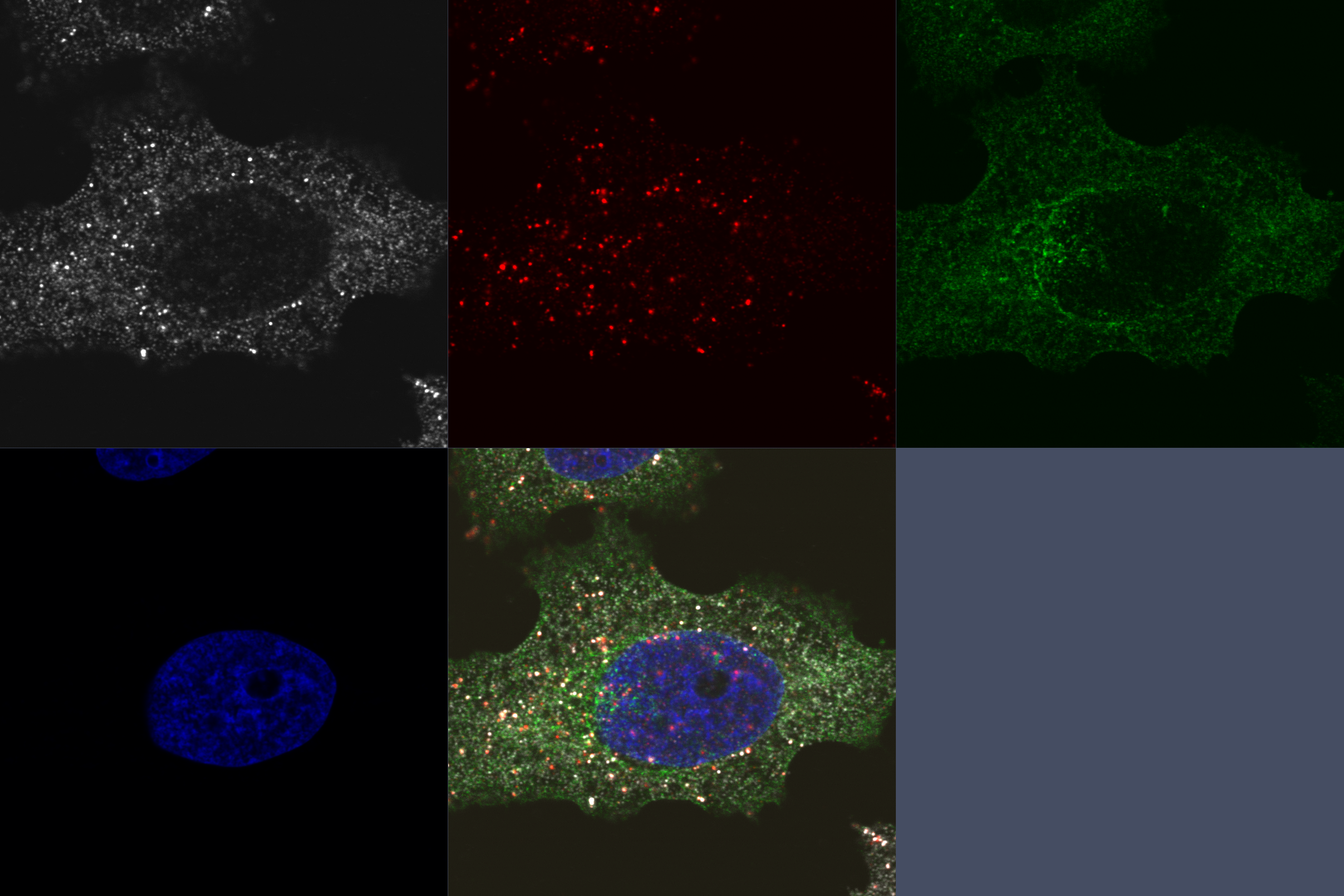

Supplement: Supplementary file 5 — Source Data for Figure 3 [file EMBJ-39-e103649-s003.zip › Figure3_Source_Data/EMBOJ-2019-103649R2-Figure_3G_right_hand_panel_Source_Data-sd.tif]

FIGURE 3A

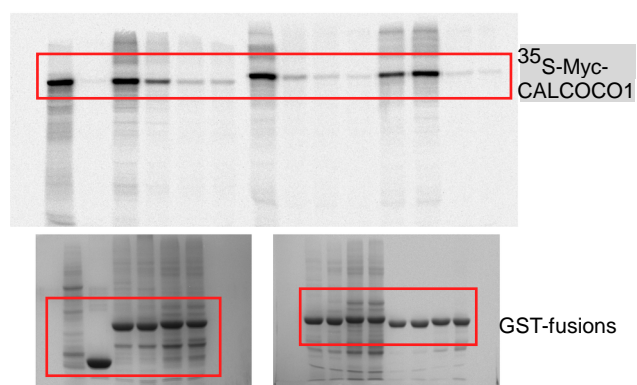

FIGURE 3B

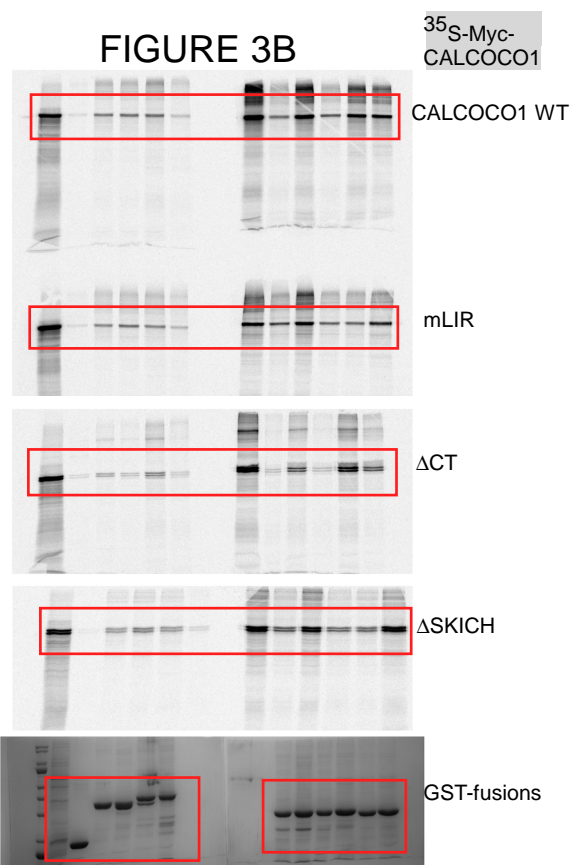

FIGURE 3C

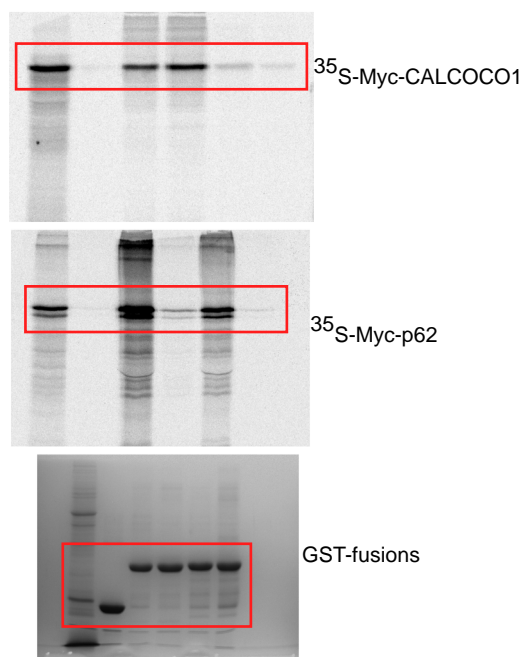

FIGURE 3D

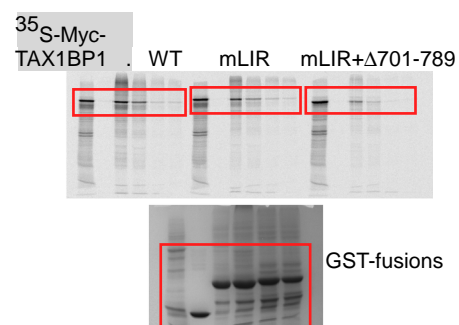

FIGURE 3E

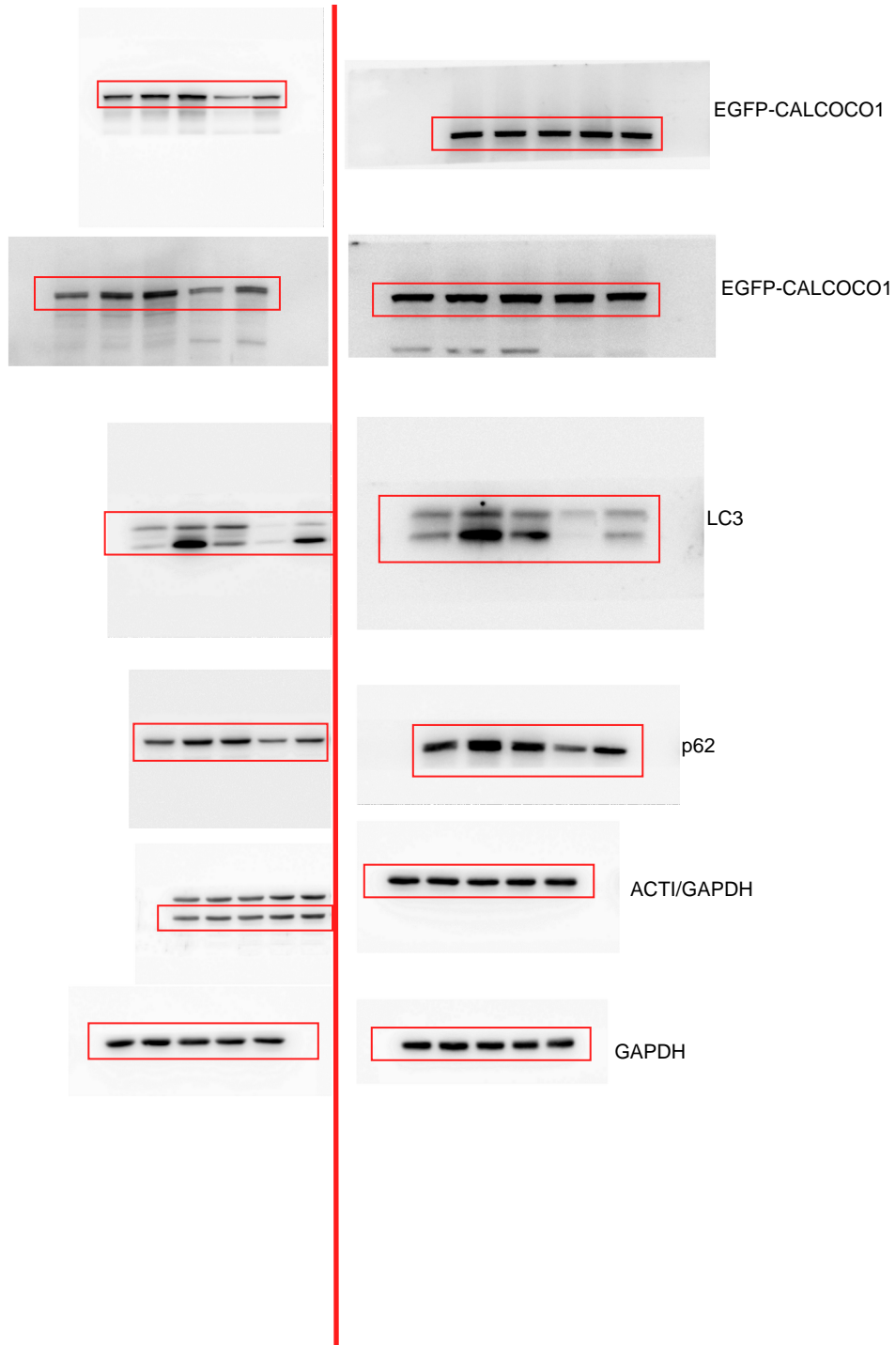

Supplement: Supplementary file 5 — Source Data for Figure 3 [file EMBJ-39-e103649-s003.zip › Figure3_Source_Data/Figure_3_Source_Data-sd.pdf]

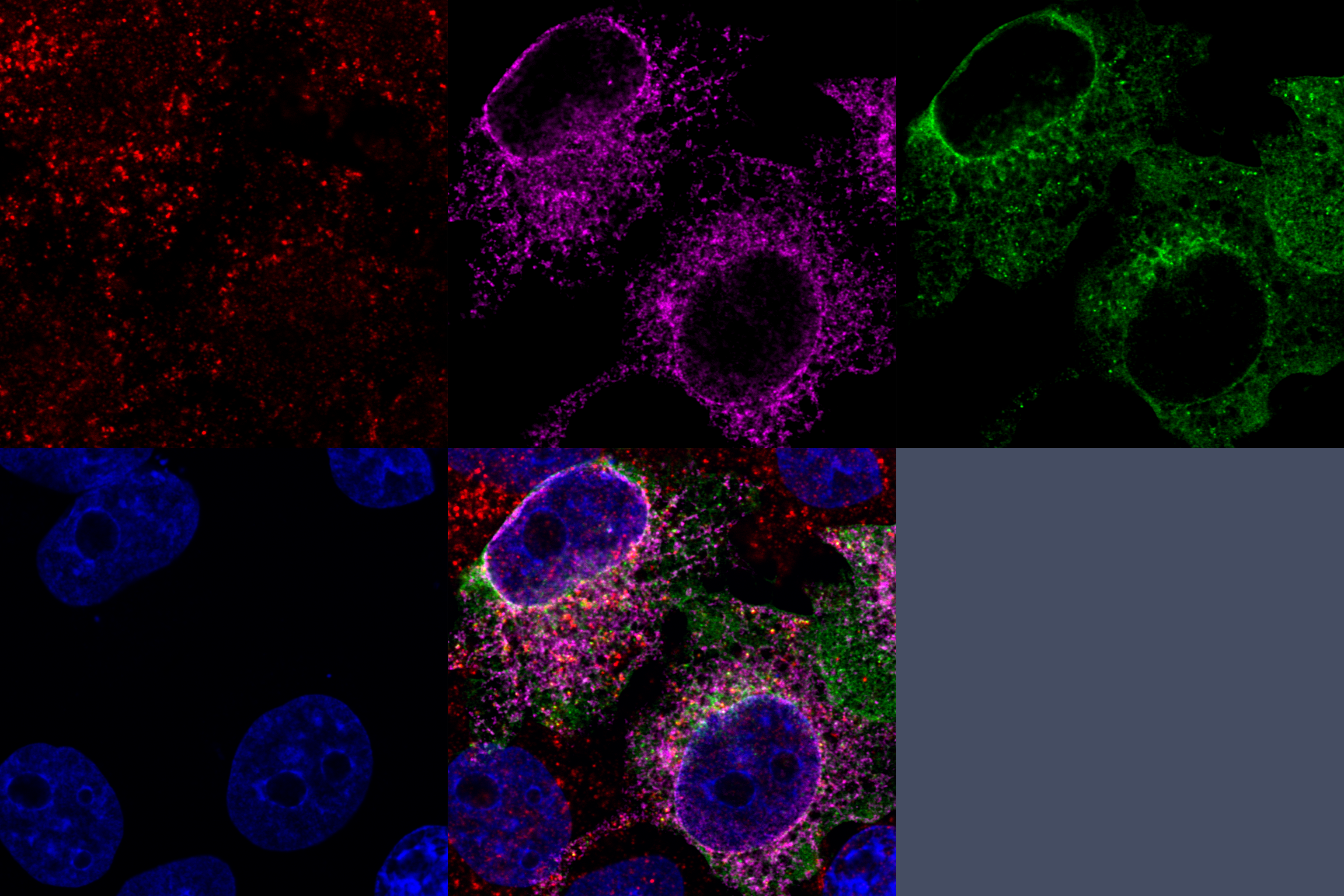

Supplement: Supplementary file 6 — Source Data for Figure 6 [file EMBJ-39-e103649-s004.zip › Figure6_Source_Data/EMBOJ-2019-103649R2-Figure_6F_lower_panel_Source_Data-sd.tif]

FIGURE 6A

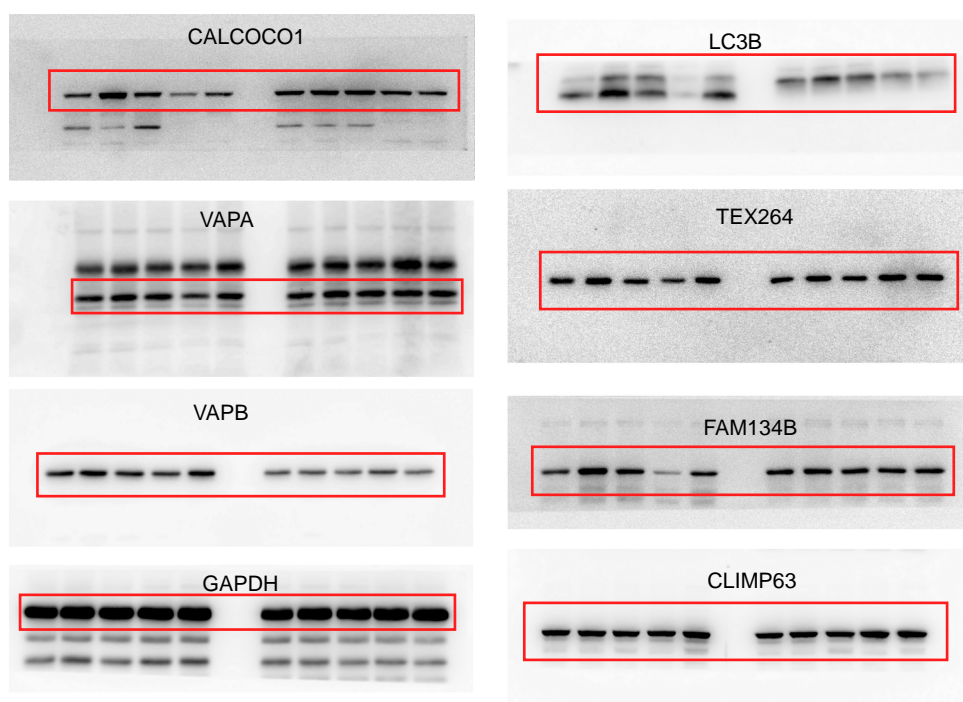

FIGURE 6C

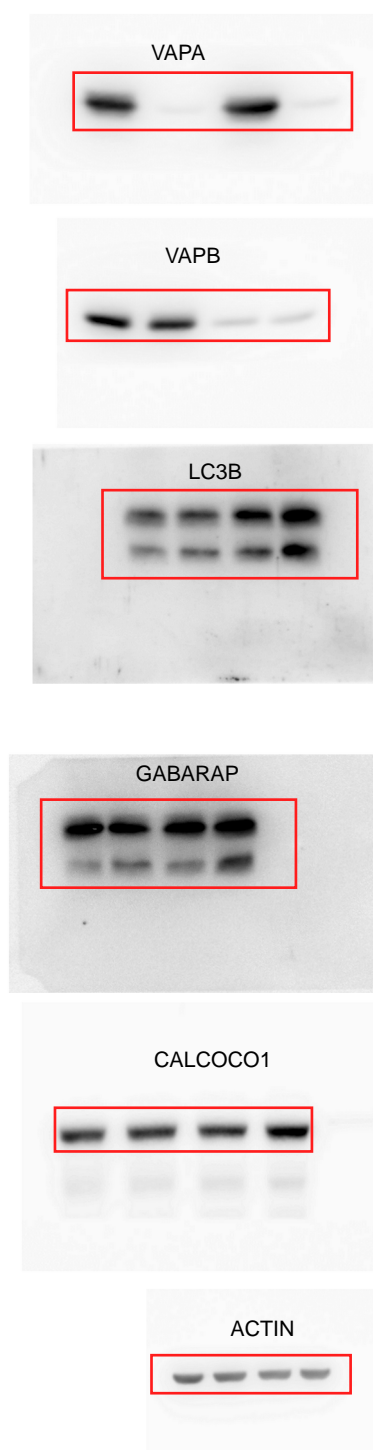

FIGURE 6D

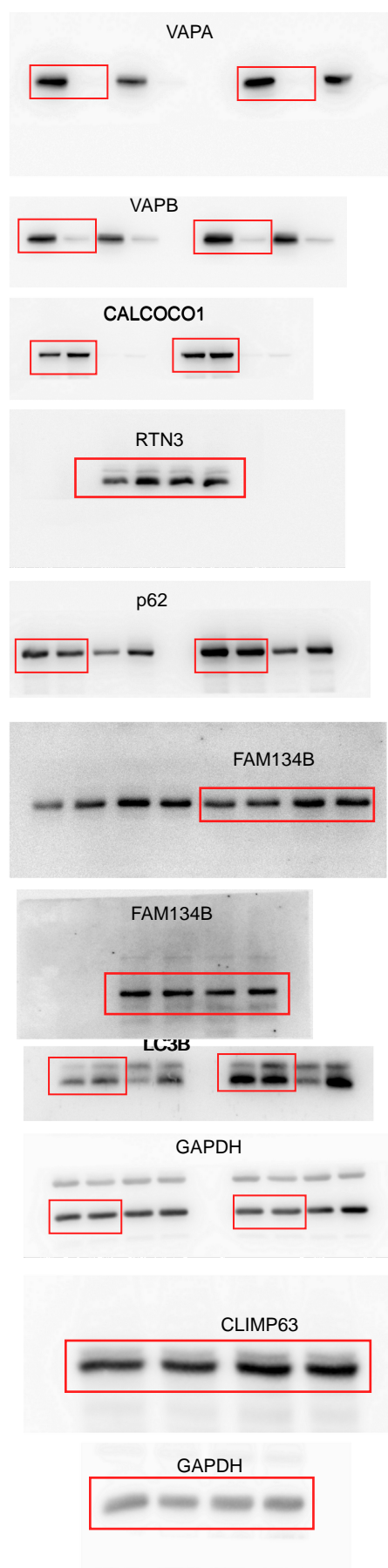

Supplement: Supplementary file 6 — Source Data for Figure 6 [file EMBJ-39-e103649-s004.zip › Figure6_Source_Data/Figure_6_Source_Data-sd.pdf]

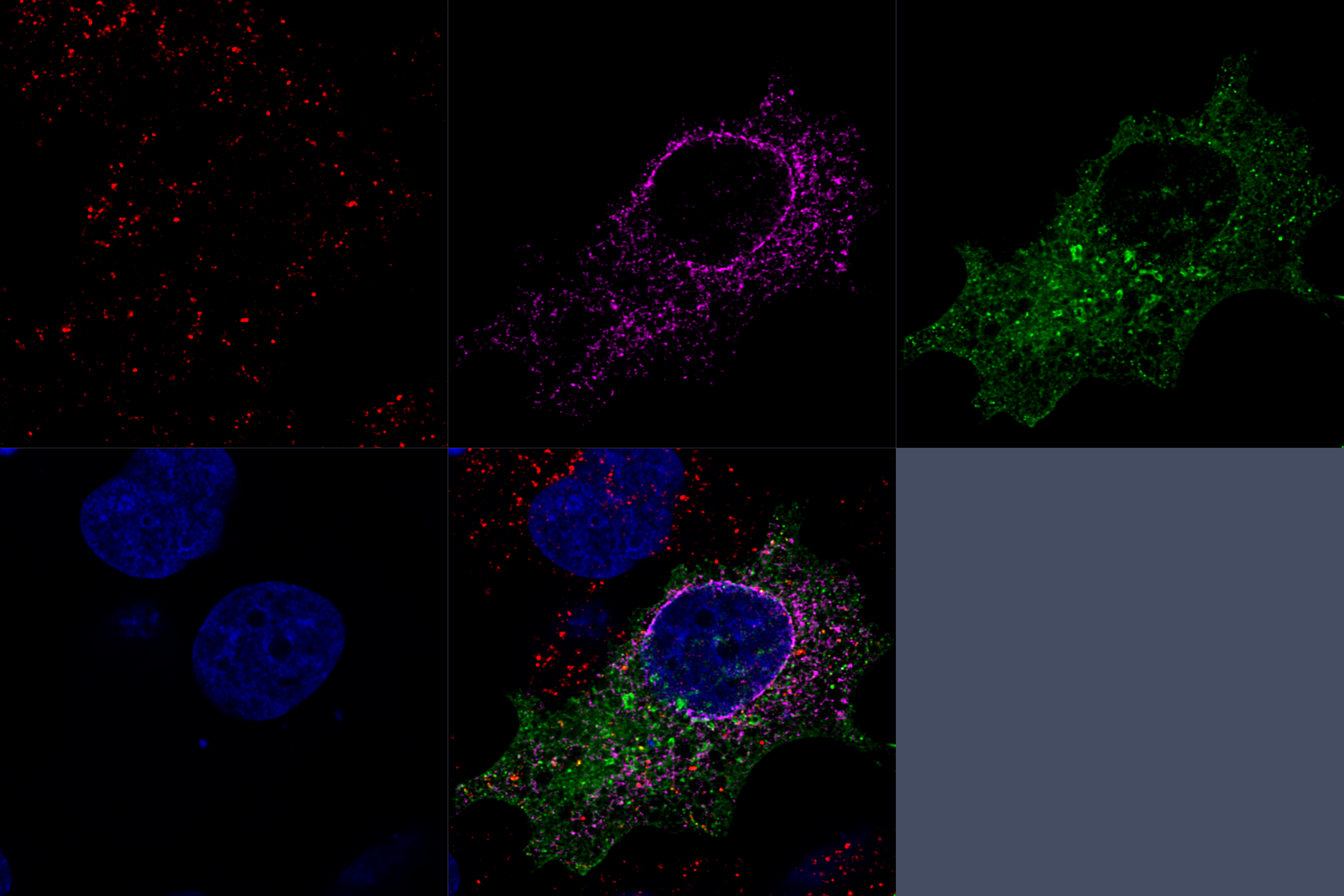

Supplement: Supplementary file 6 — Source Data for Figure 6 [file EMBJ-39-e103649-s004.zip › Figure6_Source_Data/EMBOJ-2019-103649R2-Figure_6F_upper_panel_Source_Data-sd.tif]

FIGURE 7C

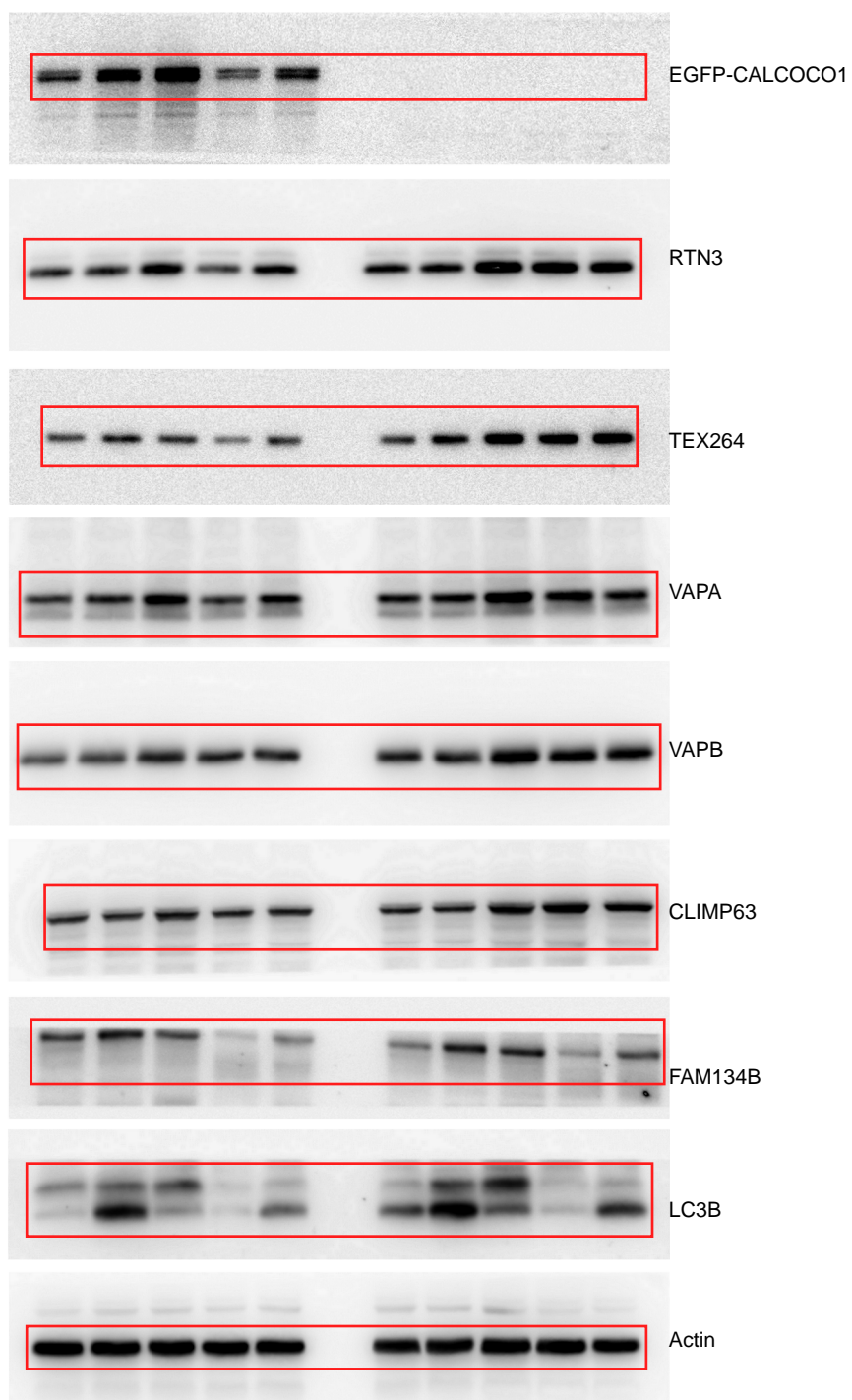

Supplement: Supplementary file 7 — Source Data for Figure 7 [file EMBJ-39-e103649-s005.pdf]

FIGURE 8A

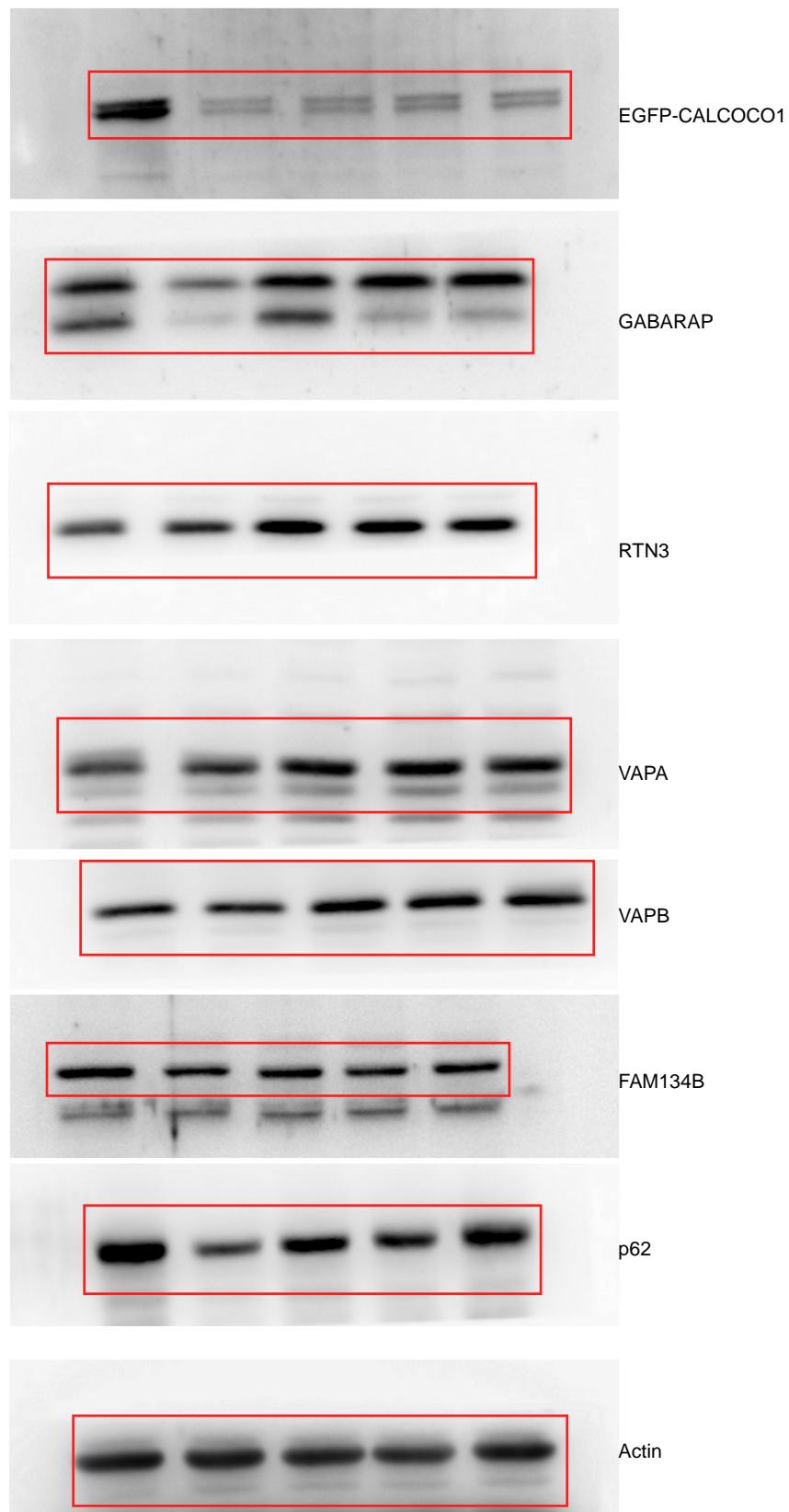

Supplement: Supplementary file 8 — Source Data for Figure 8 [file EMBJ-39-e103649-s006.pdf]
